# A visualization system for erectile vascular dynamics

**DOI:** 10.3389/fcell.2022.1000342

**Published:** 2022-10-14

**Authors:** Kota Fujimoto, Daiki Hashimoto, Kenichi Kashimada, Shinji Kumegawa, Yuko Ueda, Taiju Hyuga, Tsuyoshi Hirashima, Norimitsu Inoue, Kentaro Suzuki, Isao Hara, Shinichi Asamura, Gen Yamada

**Affiliations:** ^1^ Department of Developmental Genetics, Institute of Advanced Medicine, Wakayama Medical University, Wakayama, Japan; ^2^ Department of Plastic and Reconstructive Surgery, Wakayama Medical University, Wakayama, Japan; ^3^ Department of Molecular Physiology, National Cerebral and Cardiovascular Center Research Institute, Suita, Japan; ^4^ Department of Pediatrics and Developmental Biology, Graduate School of Medical and Dental Sciences, Tokyo Medical and Dental University, Tokyo, Japan; ^5^ Department of Urology, Wakayama Medical University, Wakayama, Japan; ^6^ Department of Pediatric Urology, Children’s Medical Center Tochigi, Jichi Medical University, Tochigi, Japan; ^7^ Mechanobiology Institute, National University of Singapore, Singapore, Singapore; ^8^ Department of Molecular Genetics, Wakayama Medical University, Wakayama, Japan; ^9^ Faculty of Life and Environmental Sciences, University of Yamanashi, Yamanashi, Japan

**Keywords:** corpus cavernosum, contraction/relaxation, vascular dynamics, erectile dysfunction, ED

## Abstract

Erection is an essential process which requires the male penis for copulation. This copulatory process depends on the vascular dynamic regulation of the penis. The corpus cavernosum (CC) in the upper (dorsal) part of the penis plays a major role in regulating blood flow inside the penis. When the CC is filled with blood, the sinusoids, including micro-vessels, dilate during erection. The CC is an androgen-dependent organ, and various genital abnormalities including erectile dysfunction (ED) are widely known. Previous studies have shown that androgen deprivation by castration results in significantly decreased smooth muscles of the CC. Experimental works in erectile biology have previously measured intracavernosal penile pressure and mechanical tension. Such reports analyze limited features without assessing the dynamic aspects of the erectile process. In the current study, we established a novel explant system enabling direct visual imaging of the sinusoidal lumen to evaluate the dynamic movement of the cavernous space. To analyze the alternation of sinusoidal spaces, micro-dissected CC explants by patent blue dye injection were incubated and examined for their structural alternations during relaxation/contraction. The dynamic process of relaxation/contraction was analyzed with various external factors administered to the CC. The system enabled the imaging of relaxation/contraction of the lumens of the sinusoids and the collagen-containing tissues. Histological analysis on the explant system also showed the relaxation/contraction. Thus, the system mimics the regulatory process of dynamic relaxation/contraction in the erectile response. The current system also enabled evaluating the erectile pathophysiology. In the current study, the lumen of sinusoids relaxed/contracted in castrated mice similarly with normal mice. These results suggested that the dynamic erectile relaxation/contraction process was similarly retained in castrated mice. However, the system also revealed decreased duration time of erection in castrated mice. The current study is expected to promote further understanding of the pathophysiology of ED, which will be useful for new treatments in the future. Hence, the current system provides unique information to investigate the novel regulations of erectile function, which can provide tools for analyzing the pathology of ED.

## Introduction

External reproductive organs are essential for the survival of the species. Among such organs, external genitalia function as copulatory devices which are under the regulation of mechanical dynamics of relaxation/contraction. When sexually stimulated, the smooth muscles of the penile corpus cavernosum are generally in a relaxed condition. Penile blood then flows into the cavernous sinus of the corpus cavernosum (CC), leading to sinusoidal expansion for erection (Hashimoto et al., 2021a; Hashimoto et al., 2021b; MacDonald and Burnett, 2021). The blood flow plays critical roles in the body by exchanging substances and supplying oxygen to cells, and this microcirculation is an essential peripheral system for erections (Belcaro et al., 2000; Bateman et al., 2003; Carlson, 2019; Guven et al., 2020; Nonomura and Hirata, 2020; Huntosova et al., 2021). The sinusoids, a microvascular system in the penis, constitute the lumen of the CC and are tubular cavities for circulating blood. The CC requires the ability to endure intense blood flow during an erection. The erectile rigidity is provided by the elasticity of the CC. The sinusoids should maintain intracavernosal pressure (ICP) during erection, but its vascular regulatory mechanism is not understood, and research on the penile corpus cavernosum and sinusoids is an important medical and biological issue (Hashimoto et al., 2021a; Hashimoto et al., 2021b).

To achieve an erection, the nerves and blood vessels should function coordinately. The sinusoids and endothelial cells within the cavernous sinus cavity release transmitters. These transmitters interact with the smooth muscles within the cavernous sinus and mediate contraction and relaxation of the cavernous sinus, leading to penile erection. Sexual stimulation excites the spinal erectile center in the sacral spinal cord, which releases acetylcholine from its nerve endings *via* the parasympathetic pelvic nerve (MacDonald and Burnett, 2021; Panchatsharam et al., 2021). This stimulation, in turn, triggers the NANC (non-adrenergic non-cholinergic) nerve, which releases nitric oxide (NO) and prostaglandin E_1_ (PGE_1_) from the endothelial cells of the sinusoids (Sheibani et al., 2022). Acetylcholine binds to G protein-coupled receptors activating phospholipase C, resulting in an increased production of inositol 1,4,5-trisphosphate (IP_3_). The increased IP_3_ activates endothelial nitric oxide (NO) synthase (eNOS), resulting in NO production. PGE_1_ activates adenylate cyclase (AC) and increases cyclic adenosine monophosphate (cAMP) production, which leads to muscle relaxation. (Ghasemi et al., 2006; Kadamur and Ross, 2013; Davies, 2015; Sheibani et al., 2022). These processes cause the blood to flow into the sinusoids, resulting in an erection. On the other hand, the sympathetic nervous system is involved in the return to the normal condition and maintenance of the detumescence of the penis. Noradrenaline released by the sympathetic nervous system causes contraction of the blood vessels and smooth muscles of the CC, resulting in the flaccid state of the penis (MacDonald and Burnett, 2021; Panchatsharam et al., 2021). Endothelin-1, a vasoconstrictor peptide produced by vascular endothelial cells, is involved in the regulation of the detumescence of the penis and influences the smooth muscle contraction of the CC (Mills et al., 2001; Mumtaz et al., 2006). Many conventional methods have been proposed to measure ICP by electrical stimulation of nerves or the contraction of penile cavernous smooth muscles. Although changes in microcirculation are considered to have significant impacts on the maintenance of biological functions, the mechanisms of erection have been revealed by mostly physiological experiments. These methods focus on physiological evaluation and their understanding through molecular analysis, and culture systems have achieved little advances (Angulo et al., 2002; Cui et al., 2018; Hashimoto et al., 2021a). We have evaluated the erection of the microcirculatory system by using a novel culture system by preparing an explant of the penile corpus cavernosum. In the current study, we establish the novel culture and visual imaging systems enabling real-time analyses to detect pathological conditions in the CC. Erectile dysfunction (ED) is the condition of insufficient erection or inability to maintain an erection. In an aging society where ED has become a serious problem and approximately one in three adult men suffer from ED (Burnett et al., 2018; Hashimoto et al., 2021a; Hashimoto et al., 2021b; Mason et al., 2022), the relationship between penile microcirculatory dysfunction and erection is still unclear. Thus, an extensive evaluation of the relaxation/contraction dynamics was further examined by using a novel parameterized ED model. Based on the systemic visualization analyses on the sinusoidal dynamics, the current system will greatly contribute to future research on the erectile responses.

## Materials and methods

### Animals

Mice were purchased from Japan SLC, Inc. (Hamamatsu, Japan). The majority of mature adult mice were of ICR strain (ranging 3–9 months of age). All mice in this study were housed under controlled temperature (21°C) with a 12:12 h light–dark cycle. The animal experiments (with Submission Number 995) were approved by the Animal Ethics Committee of Wakayama Medical University. We confirm that all the methods were carried out in accordance with relevant guidelines and regulations. The mice were anesthetized with three types of mixed general anesthesia (medetomidine, midazolam, and butorphanol) intraperitoneally (Hyuga et al., 2020). Surgical castration was performed *via* a lower abdomen incision exposing the peritoneal cavity to access the bilateral testicle. After exposing each testicle, a 7–0 Vicryl suture was used to ligate the spermatic cord, followed by cutting the vessel with scissors and then removing the testicle (Valkenburg et al., 2016). The castrated mice were left untreated for up to 8 weeks before the experiments.

### Establishment of a novel CC explant system with PB dye injection

**FIGURE 1:**
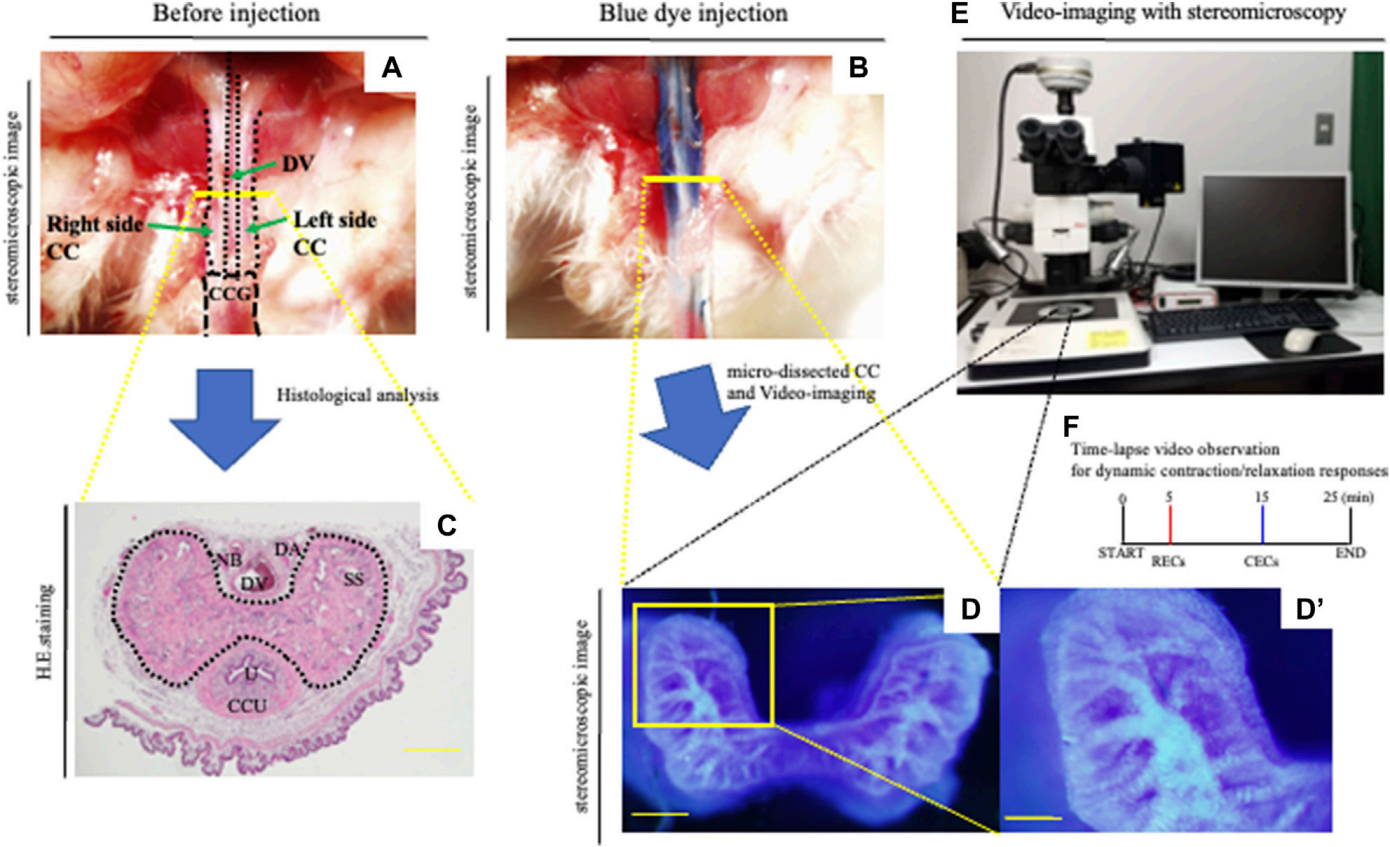
Novel *in vitro* analysis of erectile responses. **(A,B)** After the general anesthesia and being placed in the supine position, the prepuce was removed over the penis. A measure of 0.03–0.05 ml of the PB dye solution (25 mg/ml) was slowly administered left-intracavernously through the corpus cavernosum glandis (CCG) using a 29-gauge needle. **(C)** Image of hematoxylin–eosin staining of the CC. Mouse proximal penis possesses two corporal units (corpus cavernosum: CC, corpus cavernosum urethra: CCU). Many sinusoidal spaces in the CC are indicated inside the dotted line. Dorsal vein (DV), dorsal artery (DA), nerve bundle (NB), sinusoidal space (SS), and urethra (U). Scale bar 200 μm. **(D–F)** Representative images of the PB dye-stained CC and a schematic diagram of the current experiment. The mouse with PB dye injection was harvested. The CC region was isolated by microdissections removing the prepuce, dorsal vein, artery, and nerve. Subsequently, urethra and CCU were removed to generate the CC explant. The micro-dissected CC was cultured in HBSS. The area of the cytoskeleton containing collagen fibers was efficiently marked, and the dye-stained regions were observed inside the sinusoidal space. We took time-lapse pictures every 10 s to monitor the corporal tissue responses utilizing a stereomicroscope according to the observation criteria for dynamic relaxation/contraction (see Movie.1). This study is the first direct experimental system visualizing the process of relaxation/contraction in a sinusoidal space. Time-lapse video observation criteria for dynamic relaxation/contraction. Contraction-enhancing compounds; CECs (phenylephrine and endothelin-1) and relaxation-enhancing compounds; RECs (sodium nitroprusside (SNP), acetylcholine, and prostaglandin E_1_). Scale bar (D) 200 μm, **(D′)** 100 μm. All data shown are representative results of at least three independent experiments using adult specimens from different litters (*n* ≥ 3).

**FIGURE 2:**
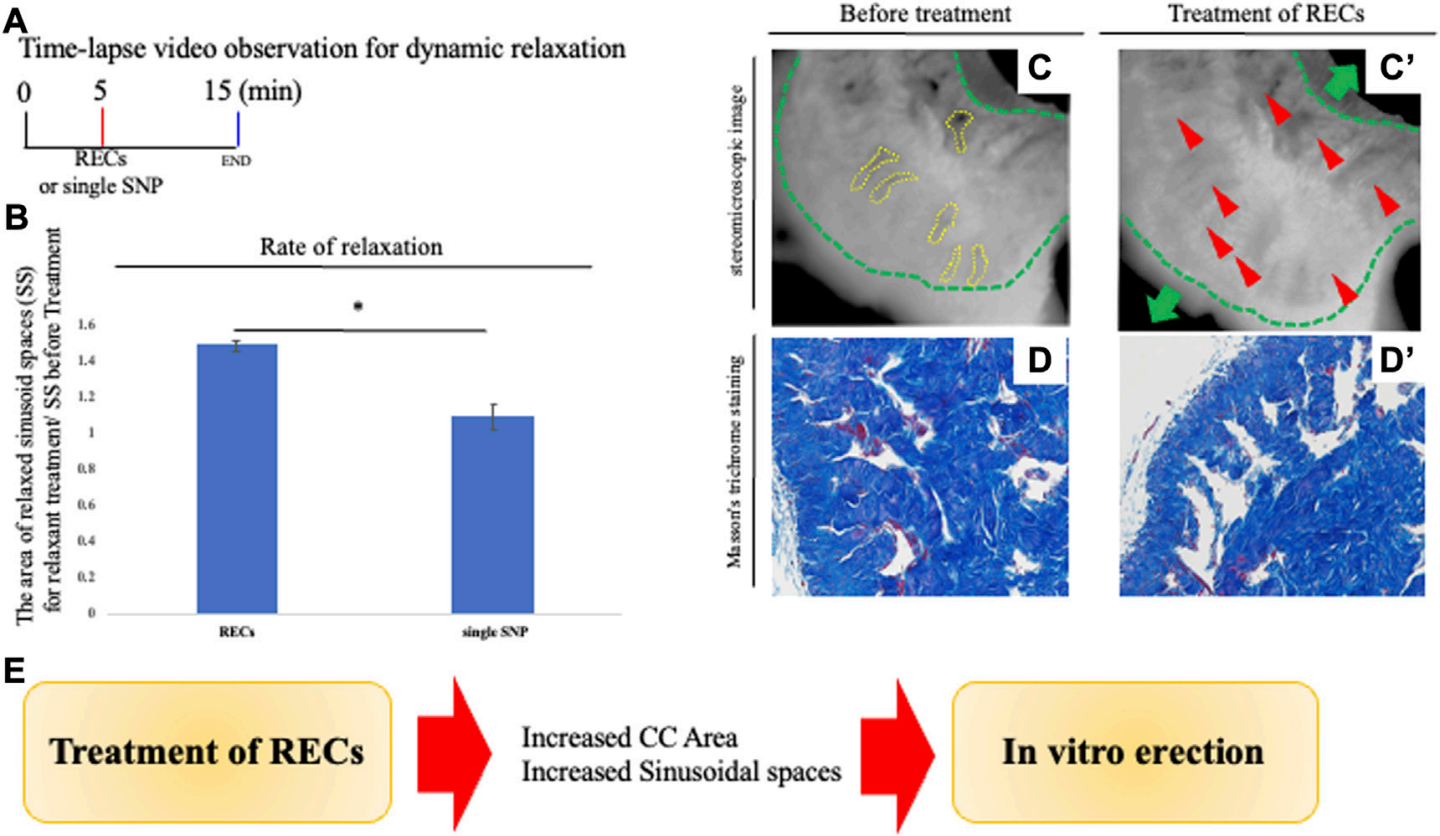
Analysis of penile relaxation responses. **(A)** Time-lapse video observation criteria for dynamic relaxation. In order to analyze the relaxation process *in vitro*, time-lapse visualization analysis (the images were taken 10 s apart) was performed. For achieving an explant relaxation after 5 min of the pre-contraction, relaxation-enhancing compounds (RECs) were applied. After REC treatment, time-lapse pictures were taken for 10 min. RECs of the final concentration of each reagent were treated with 100 μM sodium nitroprusside, 1.0 μM acetylcholine, and 10 μM prostaglandin E_1_. A time-lapse video analysis was performed by ImageJ software. **(B)** Graph was calculated from images of the movie and indicates the relative area of sinusoidal spaces by the prominent phase of the movie. The relaxed CC was significantly increased in its sinusoidal size compared with single SNP treatment. **(C,C′)** Representative 8-bit images of the PB dye-stained CC during relaxation responses. Many sinusoidal spaces in CC are indicated by yellow dotted lines. From sequential images (interval time was 10 s), the sinusoidal spaces were calculated before and after treatment by ImageJ software. Relaxation was represented as a ratio of the area of sinusoids treated by RECs and before treatment. The whole CC (indicated by the green dotted line and arrow) was extended, as well as the sinusoidal space. The red arrowheads show that the collagen area was stretched by the treatment of the RECs (see Supplementary Movie S2). Scale bar 100 μm.**(D,D′)** Image of Masson’s trichrome staining of the CC showing the collagen-rich regions. Sinusoidal spaces are separated by structures including collagen fibers (blue signal). Similar to the time-lapse images, the sinusoidal spaces were dilated after treatment with RECs, and the collagen area was extended toward the tunica albuginea. Scale bar 50 μm. **(E)** Schema shows the reproduction of the ‘erection state’ by the current system. The central collagen area near the deep arteries showed little movement while the collagen area extended outward around the adjacent collagen area. Therefore, the relaxation movement was observed to be region-dependent inside the CC. These results suggest that treatment with RECs reproduces the “erectile state” of the CC *in vitro*. All data shown are representative results of at least three independent experiments using adult specimens from different litters (n ≥ 3).

**FIGURE 3:**
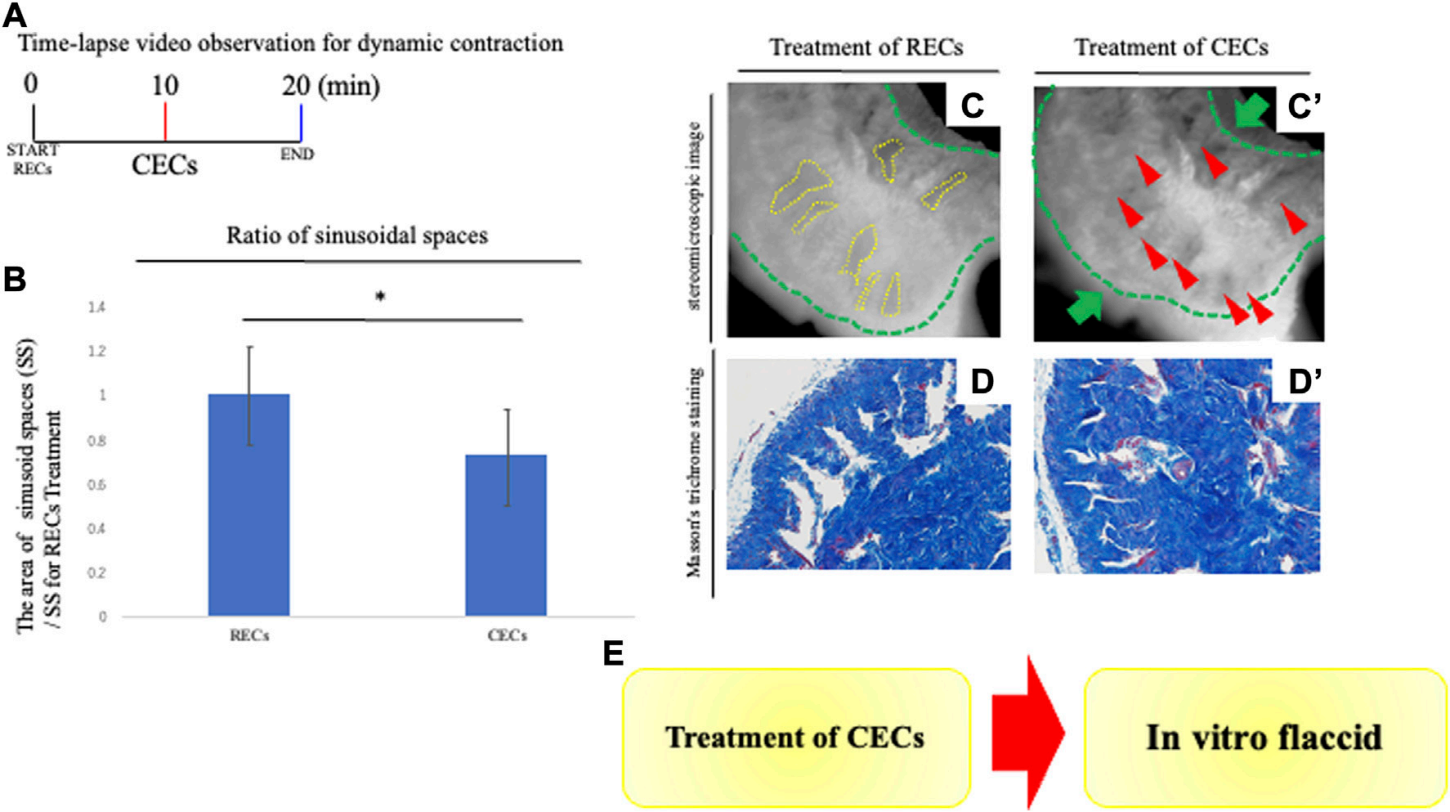
Analysis of penile contraction responses. **(A)** Time-lapse video observation criteria for dynamic contraction. For analyzing the contraction of the CC, contraction-enhancing compounds (CECs) were treated to induce vasoconstriction after 10 min of the treatment of RECs. After CECs treatment, time-lapse pictures were taken for 10 min. CECs were in the final concentration of each reagent; 1.0 μM PE and 0.1 μM endothelin-1. A time-lapse video analysis was performed by ImageJ software. **(B)** Graph was calculated from images of the movie and indicates the relative area of sinusoidal spaces by the prominent phase of the movie. The CC with treatment of CECs was significantly decreased in its sinusoidal size compared with treatment of RECs. **(C,C′)** Representative 8-bit images of the PB dye-stained CC during contraction responses. Many sinusoidal spaces in the CC are indicated by yellow dotted lines. From sequential images (interval time was 10 s), the boundaries of sinusoids were marked and the sinusoidal spaces were calculated after RECs/CECs treatment by ImageJ software. We compared the area of sinusoids at each time point (the time point for RECs treatment and CECs treatment). Contraction was represented as a ratio of the treated area of sinusoid RECs to that of CECs. The whole CC (indicated by the green dotted line and arrow) was contracted, as well as the sinusoidal space. The red arrowheads showed that the collagen area contracted by the treatment of the CECs (see Supplementary Movie S2). Scale bar 100 μm. **(D,D′)** Image of Masson’s trichrome staining of the CC showing the collagen-rich regions. Similar to the time-lapse images, the sinusoidal spaces were reduced after treatment with CECs, and the collagen area was contracted toward the central region. Scale bar 50 μm. **(E)** Schema shows the reproduction of the ‘flaccid state’ by the current system. The contractive movement was in the opposite direction to that observed by the treatment of the RECs; the expanded cavernous space prominently contracted. The extended collagen regions by the REC treatment contracted in the direction of the deep arteries. The collagen regions near the deep arteries did not show significant movement when contraction was induced by the subsequent CECs. These results suggest that the “erectile state” reproduced by the REC treatment is characterized by the return to the “flaccid state.” All data shown are representative results of at least three independent experiments using adult specimens from different litters (n ≥ 3).

The procedure used to prepare the CC explants has been partly reported previously (Hashimoto et al., 2021a). In the current study, the mice were anesthetized intraperitoneally with three types of mixed general anesthesia (medetomidine, midazolam, and butorphanol). The patent blue dye (PB dye) solution for injection in 0.9% saline at a concentration of 25 mg/ml was prepared. A measure of 0.03–0.05 ml of the PB dye solution through the corpus cavernosum glandis (CCG) using a 29-gauge needle was slowly administered left intracavernously (Figures 1A,B). The CC region was isolated by microdissections, removing the prepuce and dorsal vein, artery, and nerve. Subsequently, urethra and corpus cavernosum urethrae (CCU) were removed to generate the CC explant. The isolated explant was less than 1 mm thickness with a smooth cutting cross-sectional surface utilizing a fine scalpel for visualization analysis (Figure 1D,D’). The explant was incubated for 5 min to adhere to the grass bottom dish (Matsunami) by 0.2 μl/mm^2^ phenol red-free Matrigel matrix (354,262, Corning) to stabilize the explant for visualization. The explants were subsequently incubated in 37 C 5% CO_2_ to fix with the gel. The explant was in the Hank’s balanced salt solution (HBSS), which includes Ca^2+^ and Mg^2+^ without phenol red (14025092, Gibco) and was treated to induce pre-vasoconstriction by a final concentration of 0.1 μM phenylephrine (PE; 163-11791, Wako) after the first 5 min of the starting culture. In order to analyze the relaxation/contraction process *in vitro*, a time-lapse visualization analysis (the images were taken by each 10 s) was performed (Figure 1 E,F). For achieving explant relaxation after 5 min of the pre-contraction, relaxation-enhancing compounds (RECs) were applied (Figure 1F, Figure 2B). RECs of the final concentration of each reagent were treated; 100 μM sodium nitroprusside (SNP; PHR1423-1G, Sigma-Aldrich), 1.0 μM acetylcholine (011-00592, Wako), and 10 μM prostaglandin E_1_ (CAS 745-65-3, Cayman Chemical; Ayan et al., 1999; Moreland et al., 2000; Moreland et al., 2003; Cerqueira et al., 2008; Dalaklioglu et al., 2014; Yilmaz et al., 2014; Boydens et al., 2016; Hashimoto et al., 2021a). For analyzing the contraction of the CC, contraction-enhancing compounds (CECs) were used to induce vasoconstriction after 10 min of the treatment of RECs. CECs were in the final concentration of each reagent; 1.0 μM PE and 0.1 μM endothelin-1 (333-41981, Wako (Filippi et al., 2003; Göçmez et al., 2005; Carneiro et al., 2008; Regadas et al., 2010; Yousif et al., 2014; Hashimoto et al., 2021a)). After CECs treatment, time-lapse pictures were taken for 10 min (Figure 1F, Figure 3B). The time-lapse video analysis was performed by ImageJ software.

### Analysis of the relaxation/contraction *in vitro*

The harvested CC with PB dye staining was cultured in HBSS. RECs and CECs treatments were performed to induce relaxation/contraction. We took time-lapse pictures every 10 s to monitor the CC and sinusoid responses utilizing a stereomicroscope. From sequential images (interval time was 10 s), the boundaries of the sinusoids were marked and the sinusoidal spaces were calculated before and after RECs/CECs treatment by ImageJ software. We compared the area of sinusoids at each time point (the time point for before treatment, RECs treatment, and CECs treatment). Relaxation was represented as a ratio to the area of sinusoids treated by RECs and before treatment. Contraction was represented as a ratio of the treated area of sinusoid RECs to that of CECs. From sequential images (interval time was 10 s), the edges of the CC were defined and the time of completing the movement of contraction of the CC was calculated after CECs treatment by ImageJ software. We compared each time point of the area of the CC (control and castrated mice). The time to complete contraction in the CECs treatment was defined as the contraction time, which was evaluated as the ratio of the contraction time of CC in control to that of castrated mice.

### Histological analysis

The mouse CC tissues were fixed overnight in 4% paraformaldehyde (PFA) in phosphate-buffered saline (PBS). After that fixation, 6-μm thickness paraffin sections were prepared for hematoxylin and eosin (H.E.) staining. H.E. staining and Masson’s trichrome staining were performed by standard procedures, as previously described (Haraguchi et al., 2000; Haraguchi et al., 2001; Kajimoto et al., 2022; Ueda et al., 2022).

### Statistical analysis

For the relaxation/contraction analysis by stereomicroscopy and quantitative PCR, either the Student t-test or Welch *t*-test, followed by the F test, was performed. (values of *p* < 0.05 were considered to be significant).

## Results

### A novel system for visualizing penile vascular dynamics

An erection has been often regarded as a phenomenon of regulating blood flow to the penis. However, its mechanism holds wide ranges of phenomena. The mouse penis consists of the corpus cavernosum glandis (CCG) and the corpus cavernosum (CC) (Hashimoto et al., 2021b). The mouse penis includes the CC and foreskin, dorsal veins, arteries, nerves, and urethra (Figures 1A,C). We have constructed the penile explant experimental system with treatment of phenylephrine (PE), one of the adrenergic receptor activators and SNP (NO donor) (Hashimoto et al., 2021a). In a previous report, a two-photon excitation microscope (TPEM) was used to directly observe the contraction and relaxation processes of the sinusoids. To analyze the changes of sinusoidal spaces, micro-dissected CC explants were incubated after injection of the PB dye and examined for their structural alternations during relaxation/contraction (Figures 1B,D). When the PB dye was injected into the penis, the area of the fibrous cytoskeleton containing collagen fibers was efficiently marked and the dye-stained regions were observed inside the sinusoidal space (Figure 1D). The dynamic process of relaxation/contraction was analyzed with various external factors administered to the CC. Relaxation-enhancing compounds (RECs; combination of acetylcholine, SNP, and prostaglandin E_1_) and contraction-enhancing compounds (CECs; combination of PE and endothelin-1) were utilized to induce efficient relaxation/contraction (Figures 1B,D,D’,E,F, Supplementary Movie S1). This study is the first direct experimental system visualizing the process of relaxation/contraction in the sinusoidal space. Previous penile biology and ED studies have focused only on changes from flaccid to erection. With the treatment of CECs, the current system also evaluates alternation from erection to flaccid. The current observations demonstrate the effectiveness and novelty, which not only detects dynamic relaxation/contraction movements but also evaluates the process of erection to flaccid and the duration of erection.

### The novel explant system visualizing relaxation of the corpus cavernosum

We performed a histological analysis of the CC, the major structure of the penis containing many collagen fibers, and an internal space termed as the sinusoids of the CC. Masson’s trichrome staining was performed to analyze the corporal tissues in the CC (Perriton et al., 2002; Rodriguez et al., 2012; Hashimoto et al., 2021b; Kajimoto et al., 2022; Ueda et al., 2022). In order to visualize and evaluate the current experimental system of penile explants, analyses of the CC structure after administration of RECs were performed (Figure 2A). The lumen of sinusoids after administration of RECs was clearly dilated compared to the control. In addition, the collagen-containing tissues supporting the lumen also extended toward the tunica albuginea (Figure 2D). To analyze the dynamic movement of the cavernous space during relaxation, time-lapse video imaging was observed. The size of a sinusoid prominently expanded when RECs were applied to the CC explant (the ratio as 1.415 ± 0.055 [standard deviation (s.d.)] compared with the treatment of single SNP; Figures 2A–C; Supplementary Movie S2). The central collagen area near the deep arteries showed little movement, while the collagen area extended toward the outside around the central collagen area (Figure 2C). Therefore, the relaxation movement was observed to be region-dependent inside the CC. These results suggest that treatment with RECs reproduces the “erectile state” of the CC *in vitro* (Figure 2E).

### Analysis for dynamic contraction by the explant system

To assess the dynamic movement of the cavernous space during contraction, the treatment of RECs induced an expansion of sinusoids, followed by the treatment of CECs to induce contraction. Time-lapse video imaging was performed to observe the movement of the sinusoids (Figure 3A). The contractive movement was in the opposite direction to that observed by the treatment of the RECs; the expanded cavernous space prominently contracted (the ratio as 0.717 ± 0.122 [s.d.] compared with the treatment of RECs; Figures 3A,B; Supplementary Movie S2). The extended collagen regions by RECs treatment contracted in the direction of the deep arteries (Figure 3C). The collagen regions near the deep arteries did not show significant movement when contraction was induced by the subsequent CECs. To evaluate the efficacy of this novel system, an analysis of the dynamic structural changes during contraction was performed similar to the expansion process. Masson’s trichrome staining was performed to analyze the tissue after the CEC treatment. Compared to the RECs, it was observed that the sinusoidal lumens were reduced and the collagen-containing regions were contracted (Figure 3D).

To assess relaxation/contraction responses after the treatment of RECs and CECs, real-time PCR was performed. During erection, blood flows rapidly into the cavernous sinus, resulting in the release of nitric oxide (NO) from NO-activating cells and the relaxation of smooth muscles. Subsequently, the NO/cyclic guanosine monophosphate (cGMP) pathway induces relaxation of the cavernous smooth muscles, leading to erection. The expression of eNOS, one of the genes for NO synthase derived from endothelial cells, significantly decreased in samples with the treatment of CECs compared with the treatment of RECs (Supplementary Figure). The Rho/ROCK pathway has also been reported to be involved in the contraction of the cavernous smooth muscle and is critical for the change from erection to flaccid (Chitaley et al., 2001; Wang et al., 2002; Sopko et al., 2014). The expression of Ras homolog family member A (RhoA) genes was significantly increased after the treatment of CECs compared to the treatment of RECs (Supplementary Figure). These results suggest that the “erectile state” reproduced by the REC treatment is characterized by the return to the “flaccid state”.

### Pathological responses for relaxation/contraction

**FIGURE 4:**
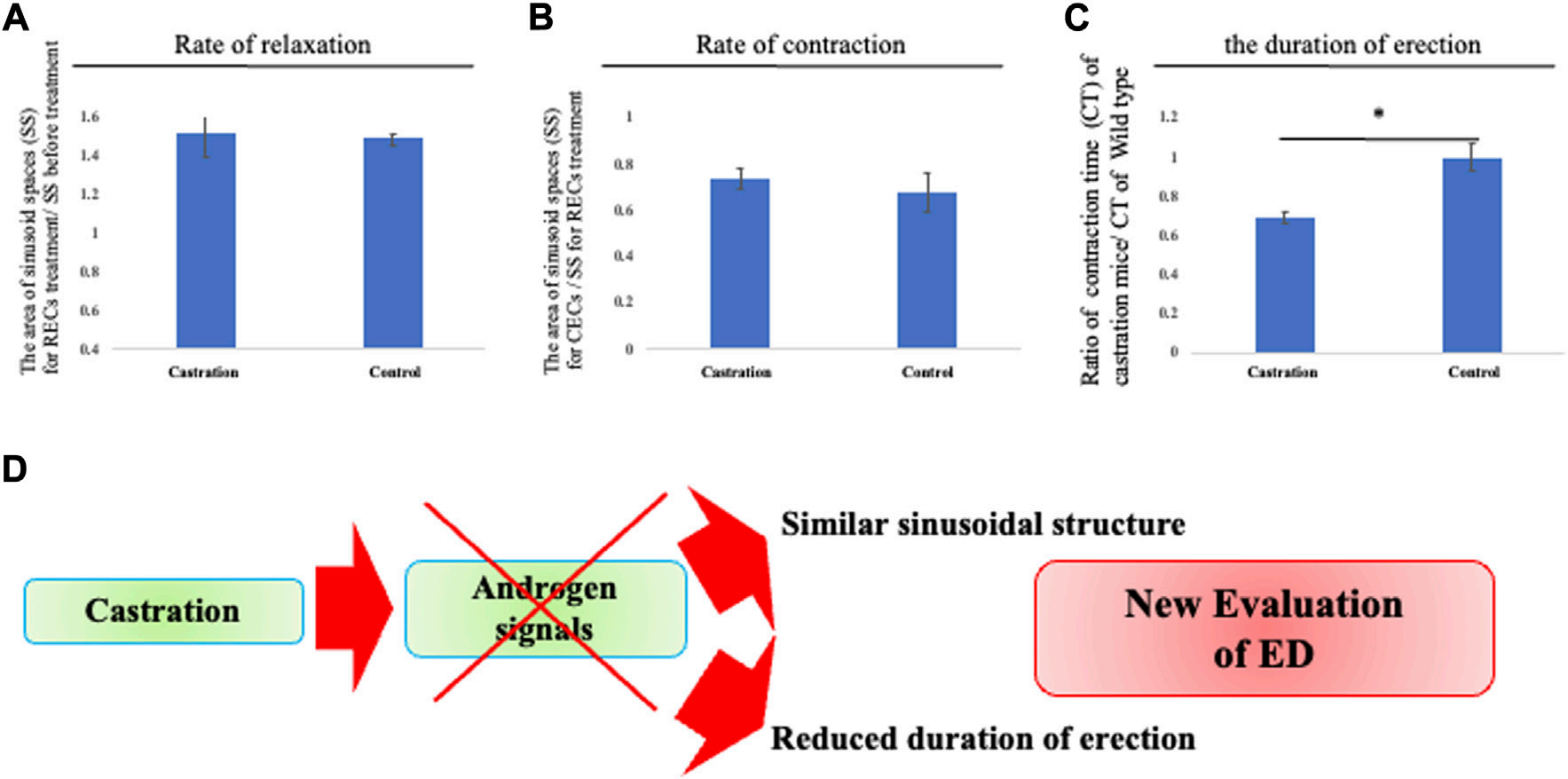
Reduced duration time of erection by castration. **(A–C)** Analysis of the relaxation/contraction *in vitro*. RECs and CECs treatments were performed to induce relaxation/contraction. From sequential images (interval time is 10 s), the edges of the CC are defined and the time of completing the movement of contraction of the CC was calculated after CECs treatment by ImageJ software. We compared the area of sinusoids at each time point (the time point for before treatment, RECs treatment, and CECstreatment). Relaxation was represented as a ratio to the area of sinusoids treated by RECs and before treatment. Contraction was represented as a ratio of the treated area of sinusoid RECs to that of CECs. The contracted/relaxed CC was not significantly altered in its sinusoidal size compared with that of the control mice. We compared each time point of the area of the CC (control and castrated mice). The time to complete contraction in CECs treatment was defined as the contraction time, which was evaluated as the ratio of the contraction time of CC in control and castrated mice. The time to contraction after treatment with CECs was shortened in castrated mice compared to control mice. **(D)** Schema shows that the androgen reduction prevents sustained erections. It is suggested that castration results in the absence of significant structural changes, stressing again the androgen-independent responses for erection. The current shortened erectile duration time suggests a reduced functional erectile capacity. Thus, the current system provides unique information to further investigate the novel regulations of erectile function and a new evaluation approach to ED.

It has been reported that castrated mice generally display erectile dysfunction (ED). In pathological conditions, previous research studies have mainly been performed on intracavernosal pressure (ICP) or histological changes of the sinusoids by castration which is considered to reduce their smooth muscles (Palese et al., 2003; Traish et al., 2003). However, the sinusoidal relaxation/contraction processes have not been directly observed. To confirm the efficacy of the current culture system for pathological evaluation, analyses of CC relaxation/contraction of castrated mice were performed. The relaxation/contraction was induced in the explant of castrated mice treated with RECs and CECs. The dynamics of the cavernous space size showed a similar relaxation/contraction rate to the control mice during relaxation/contraction (Figures 4A,B). On the other hand, when re-contraction was induced by CECs, the duration time from relaxation to contraction was markedly shortened (the ratio as 0.698 ± 0.075 [s.d.] compared with that of the control mice; Figure 4C) It has been reported that castration makes mice a model for veno-occlusive erectile dysfunction (Palese et al., 2003). These results suggest that the time from dilation to contraction mimics the duration of erection. Thus, the current model is considered as a novel evaluation system for erectile dysfunction (Figure 4D).

## Discussion

### A novel analysis of erectile responses *in vitro*

The application of a variety of animal models has contributed significantly to the advancement of research on these physiological components of penile erection. As an evaluation of erection, research has focused on the presence of an erectile response to cavernosal nerve electrical stimulation and monitoring vascular pressure and ICP pressure (Giuliano et al., 1995; Burnett, 1997; Sezen and Burnett, 2000). Alternatively, several histological evaluations of the penises of aging and diabetic patients have reported a decrease in smooth muscle cells (Shin et al., 2014; Hashimoto et al., 2021a). Such research evaluated a small part of features without analyzing the dynamic profiles of the erectile process. To assess the dynamic movement of the cavernous space, an experimental model system analyzing the sinusoidal structures related to the relaxation/contraction response was constructed with a two-photon excitation microscope (TPEM) system (Hashimoto et al., 2021a). However, the system requires specialized optical equipment and transgenic mice of actin labeling for visualization. In addition, the depth of focus is rather shallow so that only the surface region of the CC can be observed. Combined with the PB dye injection, the current system allows a clear visualization of the internal structure of the sinusoids simultaneously analyzing the relaxation/contraction responses. Furthermore, the current system reports physiological erectile responses by combining the stained CC explant by multiple mediators (RECs; relaxation-enhancing compounds including the NO donor and acetylcholine and prostaglandin E_1_, CECs; contraction-enhancing compounds including phenylephrine and endothelin-1). Blue dyes such as the PB dye have been used in medical diagnoses worldwide for decades (Manson et al., 2012). By directly staining the penis, the visual discrimination between the stained and unstained portions of the tissue was improved. Compared to the internal sinusoids as markedly stained, the collagen fibers in the tissue adjacent to the sinusoid were marked less prominently. The current system can visualize the dynamic movement of collagen fibers in the cavernous space under a stereomicroscope. The current imaging system captured, for the first time, dynamic images of collagen fibers that folded around the contracting sinusoids becoming straight during relaxation, expanding the internal sinusoidal space. The newly identified feature of this collagen movement is from adjacent the central artery to the outer tunica albuginea region. It is suggested that this movement causes the internal expansion of the sinusoid contributing significantly to erection. Furthermore, relaxation/contraction by the current multi-mediators clearly resulted in a robust erectile response compared to the single PE/SNP treatment. In the current visualization, collagen fibers adjacent to the tunica albuginea region tended to show dynamic structural changes during relaxation/contraction. Such a unique and differential movement of the central CC collagen and the surrounding collagen suggests region-dependent roles for the penile erectile responses.

### Analyses on the relaxation/contraction responses

To evaluate the advantages of the current system, the analyses for relaxation/contraction processes were performed. Erection is induced by releasing NO, which expands the CC. Acetylcholine release from parasympathetic nerve terminals increases the endothelial nitric oxide synthase (eNOS) activity; NO is also produced by neuronal nitric oxide synthase (nNOS) at the terminals of non-adrenergic and non-cholinergic cavernous nerves. The soluble guanylyl cyclase localized in cavernous smooth muscle cells is activated by NO to produce cyclic GMP with guanosine triphosphate (GTP) as a substrate. The activation of cyclic GMP-dependent protein kinase (PKG) induced by cyclic GMP leads to the relaxation of cavernous smooth muscles (Musicki and Burnett, 2006; Hashimoto et al., 2021b; Sheibani et al., 2022). Thus, the NO/cGMP pathway is essential for smooth muscle relaxation. In the current experimental system, the lumen of the sinusoid is dilated, and the collagen area is stretched after treatment of RECs. On the other hand, the lumen of the sinusoid was reduced, and the collagen area showed contraction after treatment with CECs. The expression of *eNOS* was decreased, following the treatment of CECs, suggesting that the treatment of CECs terminated erection maintenance. Several studies have demonstrated an increased nNOS production in hypoxia. Another report showed an increase in *nNOS* in the rabbit penile corpus cavernosum under ischemic conditions (Zhang et al., 1994; Ioroi et al., 1998; Wang et al., 2004). Thus, *nNOS* may not be significantly altered by the current study. Unlike other smooth muscles, which frequently change their contractile state, the smooth muscle in the CC remains contracted to maintain the flaccid state of the penis (Chitaley et al., 2001; Wang et al., 2002; Sopko et al., 2014). Contraction of the smooth muscle narrows the sinusoidal lumens, restricting the blood flow and maintaining low ICP of a flaccid penis. RhoA is present in many tissues throughout the body. The Rho-kinase activity plays essential roles in the vasoconstriction of the CC and maintenance of the flaccid state of the penis (Chitaley et al., 2001; Wang et al., 2002; Chitaley et al., 2003). *RhoA* was increased after treatment of CECs, suggesting that the CC and sinusoids were induced to contract and reproduce the flaccid state. *Rock1* and *Rock2* were not altered in the current system. ROCK has two isoforms, ROCK 1 and ROCK 2, which are involved in physiological and pathological signaling pathways (Sopko et al., 2014). ROCK is a primary downstream factor activated through RhoA (Roser et al., 2017; Zewdie et al., 2020). In addition to its regulation of cytoskeletal dynamics, they induced contractile responses in vascular smooth muscle cells (Hirooka and Shimokawa, 2005; Schofield et al., 2012; Loirand, 2015; Zewdie et al., 2020). The RhoA-ROCK activity inhibits eNOS and regulates the contractile state of the sinusoids (Chitaley et al., 2001; Chitaley et al., 2003; Sugimoto et al., 2007; Sopko et al., 2014). In transgenic mice, for sickle cell disease, Rho signaling is aberrant, particularly for the Rock2 expression, which contributes to the pathophysiology of priapism. Therefore, Rock1 and Rock2 may be involved in pathogenesis and may not prominently alter in the current system (Bivalacqua et al., 2009; Bivalacqua et al., 2010; Sopko et al., 2014; Anele et al., 2015).

In our previous reports, histological analyses of the mouse CC and sinusoids were performed (Hashimoto et al., 2021a; Hashimoto et al., 2021b; Kajimoto et al., 2022; Ueda et al., 2022). In the present experimental system, Masson’s trichrome stain, which is widely used to detect collagen fibers, was performed. The CC is one of the most significant structural components of the erectile tissue and is surrounded externally by the tunica albuginea. The CC has a complex microstructure forming a spatial network. These are formed by an extracellular matrix (ECM) of interconnected collagen and other ECMs and smooth muscle fibers (Perriton et al., 2002; Rodriguez et al., 2011; Rodriguez et al., 2012; Kajimoto et al., 2022; Ueda et al., 2022). Similar to the time-lapse images, the sinusoidal lumens expanded/contracted in the CCs treated with RECs/CECs, and the collagen around the sinusoids also elongated/contracted. Although many histological analyses of the CC have been reported so far, there have been few reports that clearly show the structural difference between erected and flaccid states. These results suggest that the current system reproduces the erectile or flaccid state *in vitro*. Hence, the current system will be evaluated in each erection/flaccid state, offering the possibility of clarifying the functions and pathology of the erectile tissue. However, erection is composed of complex mechanisms such as blood flow and nervous activities. The current system is a novel culture system utilizing the explant of the CC, and evaluation of erection focusing on the aforementioned parameters is not efficient by the current form. Administration of substances from the blood and neuronal cells should be further investigated. Further experiments and modifications are expected to be conducted utilizing the current model.

### Pathological analysis on the relaxation contraction responses

 The role of the circulatory system is to exchange oxygen, nutrients, and metabolic products and wastes from cells and organs of the body. Among them, the microcirculatory system is to primarily regulate and support the cells of the body. The functions and tissue homeostases depend on capillaries extending throughout the body. In addition, the essential roles of vascular endothelial cells for regulating such microcirculations have been recognized. It has become clear that endogenous vasoactive substances affect the relaxation/contraction of vascular smooth muscles (Belcaro et al., 2000; Bateman et al., 2003; Carlson, 2019; Guven et al., 2020). This disruption of sinusoidal microcirculation is speculated as a causative factor in tissue damage in patients with ED (Belcaro et al., 2000). ED is a common and complex disorder defined as the inability to obtain and maintain an erection sufficient for satisfactory sexual intercourse (Burnett et al., 2018). The disease has a significant impact on the quality of life and is an emerging public health problem. Since systemic vascular disease and ED share many common risk factors, it has been suggested that ED is an early marker for atherosclerosis, cardiovascular events, and asymptomatic systemic vascular diseases (Chien and Schwarz, 2007; Imprialos et al., 2021). Androgens play an important role in the maintenance of the erectile tissue structure, smooth muscle, and endothelium (Hyuga et al., 2019; Kajimoto et al., 2022; Ueda et al., 2022). Previous studies have shown that the influx of blood into the penile corpus cavernosum increases ICP. Testosterone-treated castrated animals have higher ICP than castrated animals, indicating that erection is an androgen-dependent process (Palese et al., 2003). To evaluate the current system with pathological conditions, time-lapse imaging was performed in castrated mice. No prominent differences were observed in the rate of relaxation/contraction of the sinusoidal spaces compared to those of the control mice.

The novelty of the current system includes the time to contraction mimics the duration of erection, allowing for the assessment of erection duration *in vitro*. Castrated mice possessed a shorter erectile duration time than the control mice, which displayed an inability to maintain an erection. These results are consistent with reports that the neuroelectric stimulation of chronically castrated animals increases ICP and that erection is not completely lost in those mice (Mills et al., 1994). It is suggested that castration results in the absence of significant structural changes, stressing again the androgen-independent responses for erection. Castration is considered to cause veno-occlusive erectile dysfunction (Palese et al., 2003), and the current shortened erectile duration time suggests a reduced functional erectile capacity. Thus, the current system provides unique information to further investigate the novel regulations of erectile dysfunction.

## Data Availability

The original contributions presented in the study are included in the article/Supplementary files, further inquiries can be directed to the corresponding author.
