# Supplementary figures and images for: A visualization system for erectile vascular dynamics
Source: Front Cell Dev Biol. 2022 Oct 14;10:1000342. doi: 10.3389/fcell.2022.1000342 (PMC9615422; doi:10.3389/fcell.2022.1000342)

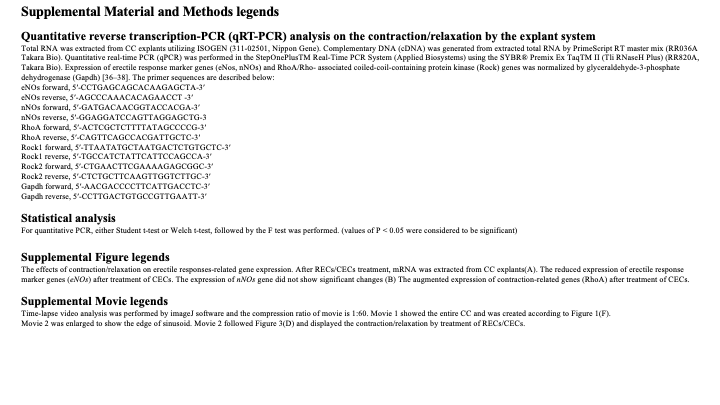

Supplement: Supplementary file 1 [file Image1.TIFF]

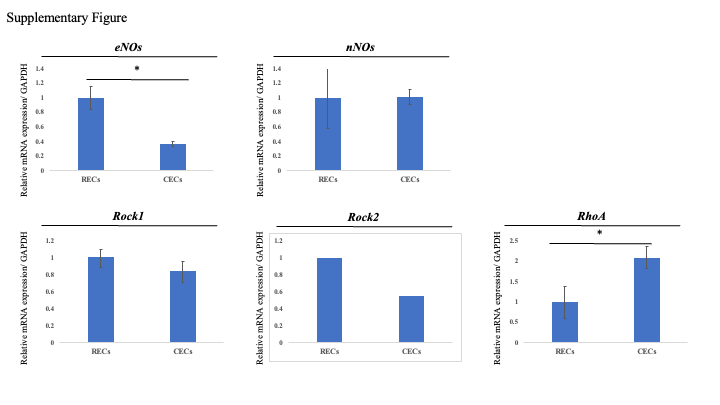

Supplement: Supplementary file 3 [file Image2.TIFF]
